# Long‐term trends in metabolic profiles across Chinese birth cohorts: A real‐world big data analysis

**DOI:** 10.1002/ctm2.70660

**Published:** 2026-06-17

**Authors:** Ying Xiong, Xincen Duan, Jing Zhu, Jiayi Huang, Baishen Pan, Wenqi Shao, Beili Wang, Wei Guo

**Affiliations:** ^1^ Department of Laboratory Medicine Zhongshan Hospital Fudan University Shanghai China; ^2^ Department of Laboratory Medicine Geriatric Medical Center Shanghai China; ^3^ Zhongshan Hospital (Xiamen) Fudan University Xiamen China; ^4^ Department of Laboratory Medicine Wusong Branch Zhongshan Hospital Fudan University Shanghai China

## Abstract

**Background:**

The global burden of metabolic diseases is increasingly severe, with a continuous rise in prevalence among younger populations. There is an urgent need to explore the long‐term trends of metabolic risk factors across generations. This study aimed to investigate birth cohort effects on metabolic profiles among Chinese adults to provide a basis for precision prevention and control.

**Methods:**

This retrospective longitudinal study analysed real‐world clinical laboratory data (2010–2024) from Fudan University Zhongshan Hospital in China including five metabolic indicators—fasting glucose (FG), total cholesterol (TC), triglycerides (TG), low‐density lipoprotein cholesterol (LDL‐C) and high‐density lipoprotein cholesterol (HDL‐C). The study included 3 888 861 adults born between 1930 and 1999, with 9059 individuals forming a fixed cohort for annual testing. Birth cohorts were defined in 10‐year intervals from the 1930s to the 1990s. Linear regression (overall population) and mixed‐effects models (fixed cohort) were used to assess cohort trends, adjusting for age and sex.

**Results:**

In the overall population, compared with earlier birth cohorts, later cohorts had significantly higher levels of FG (1990s vs. 1930s: +.438 mmol/L; *p* < .001). Lipid changes were more complex: TC and LDL‐C decreased in earlier cohorts (1930s‒1970s) but rebounded in the 1980s‒1990s (LDL‐C [1990s vs. 1930s]: +.034 mmol/L; *p* < .001), while HDL‐C and TG continued to decline (HDL‐C [1990s vs. 1930s]: ‒.075 mmol/L; TG [1990s vs. 1930s]: ‒.218 mmol/L; *p* < .001). Additionally, a fixed cohort with repeated annual measurements was established to provide longitudinal validation, enhancing the robustness of findings against population variability.

**Conclusions:**

The significant birth cohort effects on metabolic indicators suggest that younger generations, particularly those 1980s‒1990s cohorts, face greater metabolic risks than 1930s‒1970s cohorts. These findings support intensifying early screening of blood glucose and lipid profiles and encourage prompt dietary and lifestyle modifications to counter the rising metabolic trends in the 1980s–1990s cohorts.

**Key points:**

- Younger generations (1980s–1990s) exhibit higher fasting glucose and rebounding total cholesterol and low‐density lipoprotein cholesterol, alongside declining high‐density lipoprotein cholesterol and triglycerides compared to 1930s–1970s cohorts.
- A large fixed cohort with longitudinal follow‐up robustly confirms the accelerated metabolic risk accumulation in younger Chinese generations.
- Findings underscore the critical need for early screening and preventive strategies targeting the 1980s–1990s birth cohorts.

## Graphical abstract

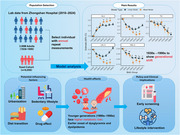

## 1 INTRODUCTION

With the changes in lifestyle and the acceleration of population aging, the disease spectrum of Chinese residents has shifted significantly, with a rapid increase in the prevalence of chronic diseases such as cardiovascular diseases and diabetes.^1^ The prevalence of cardiovascular diseases in China increased from 4.2% in 1990 to 8.5% in 2019, a rise of 99.75%.^2^ The prevalence of type 2 diabetes in China increased from 9.7% in 2007 to 11.9% in 2019.^3^ However, an increasing number of studies demonstrated a concerning trend of earlier onset age for chronic diseases. In China, the prevalence of type 2 diabetes in people under 40 years old increased the fastest from 2008 to 2017, rising from 3% to 10.9%.^4^ Meanwhile, from 2010 to 2019, the prevalence of dyslipidaemia in China increased from 28.6% to 32.8%, with the most significant increase in the 30‒39 years age group.^5^ There is also a trend of younger onset age for acute coronary syndrome and higher proportions of complicated cardiovascular diseases.^6^

Abnormal fasting glucose (FG) and dyslipidaemia are important risk factors for type 2 diabetes and cardiovascular diseases, which are closely related to disease onset and prognosis.^7^, ^8^ Previous studies, such as the US NHANES study (1999‒2020), have used cross‐sectional data to construct birth cohorts and found population‐level improvements in total cholesterol (TC) and triglyceride (TG) levels decelerated and adverse trends in glucose levels accelerated in more recent birth cohorts.^9^ However, such kinds of cross‐sectional studies lacked repeated measurements in the same participants, and were unable to assess individual‐level changes in cardiovascular risk factors over time. Also, the birth cohort effects of metabolic indicators in developing countries undergoing rapid nutritional transitions may differ due to different dietary transitions and public health policies, and the metabolic changes of this type of people have not yet been studied.

Therefore, this study analysed real‐world large‐scale laboratory testing data from 2010 to 2024 in China to statistically investigate the trends in five core metabolic indicators, FG, TC, TG, low‐density lipoprotein cholesterol (LDL‐C) and high‐density lipoprotein cholesterol (HDL‐C), among different birth cohorts. Unlike previous cross‐sectional studies, this study leverages both a large cross‐sectional sample and a longitudinal fixed cohort to capture both population‐level and individual‐level trends, providing a more comprehensive view of metabolic shifts in a rapidly transitioning population. The aim was to reveal the intergenerational patterns of metabolic changes in China over the past 15 years and provide evidence‐based support for the precision prevention and control of cardiovascular diseases and diabetes.

## 2 METHODS

### 2.1 Study design and population

**FIGURE 1:**
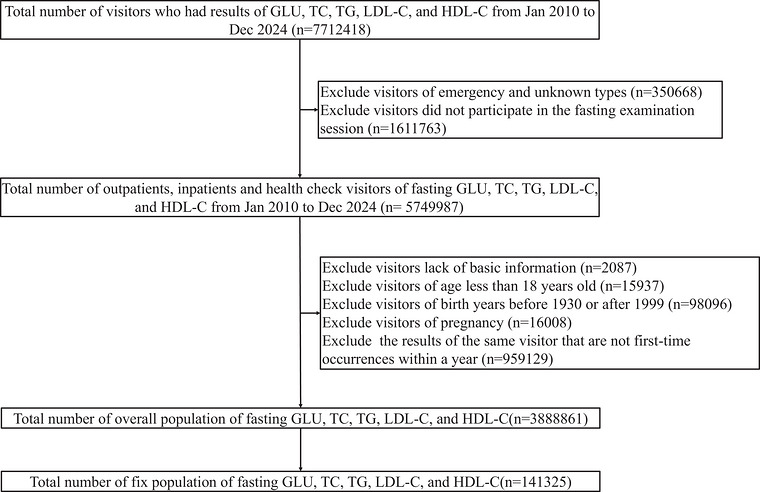
Sample inclusion and exclusion flowchart.

The data were collected from individuals who underwent laboratory tests for FG, TC, TG, HDL‐C and LDL‐C extracted from the Laboratory Information System (LIS) of Fudan University Zhongshan Hospital from 1 January 2010 to 31 December 2024. The overall population included adults born between 1930 and 1999. The following exclusion criteria were applied: (1) emergency patients, (2) non‐fasting state during testing, (3) incomplete basic information, (4) age under 18 years and (5) pregnancy. To ensure that the data distribution was not affected by repeated testing, only the first test result of each year was analysed for each participant. Since the number of adults born between 2000 and 2009 was relatively small during the study period, this group was not included in the analysis. A fixed cohort was selected from the overall data, consisting of individuals who had at least one FG test in our hospital each year (Figure 1). The fixed population includes all individuals who met the requirements during the period from 2010 to 2024. The study was approved by the Ethics Committee of Fudan University Zhongshan Hospital (B2023‐363).

### 2.2 Laboratory measurements

All tests in this study used serum samples. Participants had 5 mL of venous blood drawn in the morning (6:00‒10:00) after fasting for at least 10 h. The samples were collected in tubes with inert separation gel and centrifuged at 2000× *g* for 10 min to separate the serum. FG was measured using the glucose oxidase method with a Hitachi 7600 automated analyser and reagents from Shanghai Kehua Bio‐Engineering Co., Ltd., from 2005 to 2016, and using the hexokinase method with a Roche cobas 8000 c702 automated biochemical analyser and its reagents from 2017 onwards. The performance validation was passed during the change of the testing system. During the study period, the annual coefficient of variation (CV) for glucose internal quality control materials at high, medium and low levels remained consistently around 2%. Lipid indicators (TC, TG, HDL‐C and LDL‐C) were measured using a Roche Diagnostics (Switzerland) automated biochemical analysis system and its reagents, all by homogeneous enzyme colorimetric method. LDL‐C was calculated using the Friedewald equation when TG ≤4.5 mmol/L^10^ and measured directly by Roche homogeneous enzyme colorimetric method when TG >4.5 mmol/L. The laboratory used the Westgard multi‐rule quality control rules, with a frequency of one quality control run every 8 h during sample testing to ensure the stability of the testing data. The laboratory participated in external quality assessment programs of the National Health Commission and the Shanghai Center for Clinical Laboratory annually, with satisfactory results.

### 2.3 Statistical analysis

The birth year was calculated by subtracting the age from the year of testing, consistent with previous publications.^9^ Birth cohorts were defined in 10‐year intervals (1930s/1940s/1950s/1960s/1970s/1980s/1990s). Each cohort's age range was from 18 years to the maximum age (e.g., the 1960s cohort was aged 50‒59 years in 2010 and 64‒73 years in 2024). Due to the small sample size of the 1990s birth cohort in the fixed population (*n* < 30), it was not analysed separately. First, the unadjusted means of FG and lipids for each 10‐year birth cohort were calculated at different ages during the observation period, with TG geometric means calculated.

We employed two primary analytical approaches to assess secular trends: one for the overall cross‐sectional population and one for the fixed longitudinal cohort.

1. Overall population analysis: for the cross‐sectional data, we used linear regression models to estimate the association between continuous birth year (the main predictor) and each metabolic outcome (FG, TC, TG, LDL‐C and HDL‐C). TG levels were natural‐log‐transformed to approximate a normal distribution prior to analysis, and geometric means are reported. The basic form of the model was:

*Y_𝑖 = β*_0_ + *β*_1_* (BirthGroup_𝑖)+*β*_2_ * (Age_𝑖) + *β*_3_ * (Sex_𝑖) + *β*_4_ * (ParticipantType_𝑖) + *β*_5_ * (Age_𝑖 * Sex_𝑖) + 𝜀_𝑖

 where *Y*_*i* is the metabolic outcome for individual *i*, Age_*i* is the age at testing (modelled as a continuous variable for FG and as a categorical variable in 10‐year strata for lipids that revealed non‐linear relationships), Sex_*i* is sex (male/female), ParticipantType_*i* is a binary variable (health check‐up or patient) and Age_*i* * Sex_*i* is an interaction term.

1. Fixed cohort analysis: for the longitudinal subset of individuals with repeated measurements, we used linear mixed‐effects models to account for within‐subject correlation. The model included a random intercept for each participant:

*Y*_𝑖*j* = *β*_0_ + *β*_1_ * (BirthGroup_i) + *β*_2_ * (Age_𝑖*j*) + *β*_3_ * (Sex_𝑖) + *β*_4_ * (ParticipantType_𝑖) + *β*_5_ * (Age_𝑖 * Sex_𝑖) + *b*_𝑖 + 𝜀_𝑖*j*

where *Y*_*ij* is the measurement for individual *i* at time *j*, *b*_*i* is the random intercept for individual *i*, and other terms are as defined above.

All analyses were completed using R 4.3.1. The mixed‐effects models were fitted using the lme4 package (version 1.1.37). A two‐sided *p*‐value <.05 was considered statistically significant.

## 3 RESULTS

### 3.1 Overall characteristics

A total of 3 888 861 individuals were included in the overall population, with a median age of 54 years (interquartile range [IQR] 40‒65); 1 684 416 (43.3%) were women and 2 204 445 (56.7%) were men; 2 221 628 (57.1%) were patient and 1 667 233 (42.9%) were health check‐ups. The fixed cohort included 9059 individuals, with a median age of 49 years (IQR 37‒59) in the first year; 3958 (43.7%) were women and 5101 (56.3%) were men; 65 453 (46.3) were patient and 75 872 (53.7) were health check‐ups. The specific distribution of the number of tests for each item is shown in Tables 1 and S1.

**TABLE 1:** Characteristics of the study population of different types of participants.

	Glucose		Total cholesterol		Low‐density lipoprotein cholesterol		High‐density lipoprotein cholesterol		Triglycerides		All	
	Overall population	Fixed cohort	Overall population	Fixed cohort	Overall population	Fixed cohort	Overall population	Fixed cohort	Overall population	Fixed cohort	Overall population	Fixed cohort
*N*	3 025 075	5993	2 698 647	4239	2 447 960	2843	2 446 064	2829	2 699 022	4240	3 888 861	9059
PatientAge, median (IQR)	52 (38, 64)	47 (35, 58)	52 (38, 63)	49 (37, 59)	53 (40, 64)	54 (42, 63)	53 (40, 64)	54 (42, 63)	52 (38, 63)	49 (37, 59)	54 (40, 65)	49 (37, 59)
Birth_groups												
1930‒1939	135 319 (4.5)	379 (6.3)	111 256 (4.1)	320 (7.5)	107 000 (4.4)	310 (10.9)	106 844 (4.4)	310 (11.0)	111 258 (4.1)	320 (7.5)	179 325 (4.6)	702 (7.7)
1940‒1949	345 395 (11.4)	809 (13.5)	290 392 (10.8)	640 (15.1)	281 262 (11.5)	575 (20.2)	280 837 (11.5)	573 (20.3)	290 424 (10.8)	640 (15.1)	464 609 (11.9)	1347 (14.9)
1950‒1959	675 074 (22.3)	1351 (22.5)	592 903 (22.0)	1053 (24.8)	557 465 (22.8)	775 (27.3)	556 830 (22.8)	767 (27.1)	592 966 (22.0)	1051 (24.8)	910 813 (23.4)	2238 (24.7)
1960‒1969	615 860 (20.4)	1244 (20.8)	554 089 (20.5)	858 (20.2)	512 643 (20.9)	542 (19.1)	512 290 (20.9)	538 (19.0)	554 158 (20.5)	859 (20.3)	794 628 (20.4)	1869 (20.6)
1970‒1979	496 447 (16.4)	1307 (21.8)	457 592 (17.0)	835 (19.7)	412 393 (16.8)	419 (14.7)	412 194 (16.9)	419 (14.8)	457 608 (17.0)	835 (19.7)	618 716 (15.9)	1729 (19.1)
1980‒1989	527 855 (17.4)	898 (15.0)	493 561 (18.3)	530 (12.5)	402 709 (16.5)	219 (7.7)	402 622 (16.5)	219 (7.7)	493 707 (18.3)	532 (12.5)	635 863 (16.4)	1166 (12.9)
1990‒1999	229 122 (7.6)	‒	198 852 (7.4)	‒	174 487 (7.1)	‒	174 446 (7.1)	‒	198 899 (7.4)	‒	284 904 (7.3)	‒
Sex												
Female	1 299 016 (42.9)	2570 (42.9)	1 158 408 (42.9)	1850 (43.6)	1 073 501 (43.9)	1273 (44.8)	1 072 396 (43.8)	1264 (44.7)	1 158 550 (42.9)	1850 (43.6)	1 684 416 (43.3)	3958 (43.7)
Male	1 726 059 (57.1)	3423 (57.1)	1 540 239 (57.1)	2389 (56.4)	1 374 459 (56.1)	1570 (55.2)	1 373 668 (56.2)	1565 (55.3)	1 540 472 (57.1)	2390 (56.4)	2 204 445 (56.7)	5101 (56.3)

*Note*: The data of the fixed cohort were collected in the first year.

Abbreviation: IQR, interquartile range.

### 3.2 Birth cohort effects on fasting glucose levels

**FIGURE 2:**
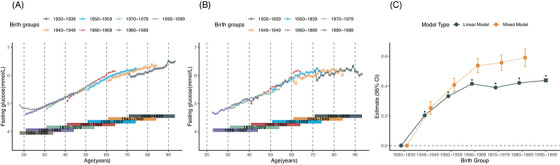
Analysis of fasting glucose levels with age and model analysis across different birth cohorts.

In the overall population, individuals from later birth cohorts had higher FG levels than those from earlier birth cohorts. The unadjusted mean trend of FG with age showed that later birth cohorts had higher mean levels at the same age: at the age of 71 years, the mean FG levels for the 1930s, 1940s and 1950s birth cohorts were 6.05 (1.92), 6.10 (1.84) and 6.18 (1.95) mmol/L, respectively; while at the age of 31 years, the mean levels for the 1970s, 1980s and 1990s birth cohorts were 4.82 (.86), 4.94 (.88) and 4.91 (.90) mmol/L, respectively (Figure 2A). After adjustment by linear regression model (with the 1930s birth cohort as the reference group), the increase in FG gradually increased with later birth cohorts, reaching the highest value in the 1990s birth cohort (1940s: +.202 mmol/L; 1950s: +.332 mmol/L; 1960s: +.415 mmol/L; 1970s: +.390 mmol/L; 1980s: +.421 mmol/L; 1990s: +.438 mmol/L, all *p* <.001) (Figure 2C).

The fixed population further confirmed this pattern. The unadjusted mean trend of FG with age in the fixed population was consistent with the overall population, with later birth cohorts still having higher unadjusted mean levels of FG at the same age (Figure 2B). After adjustment by mixed‐effects model, the increase in FG with later birth cohorts was even higher than that in the overall population (1940s: +.252 mmol/L; 1950s: +.407 mmol/L; 1960s: +.537 mmol/L; 1970s: +.556 mmol/L; 1980s: +.589 mmol/L, all *p* <.001) (Figure 2C).

### 3.3 Birth cohort effects on total cholesterol levels

**FIGURE 3:**
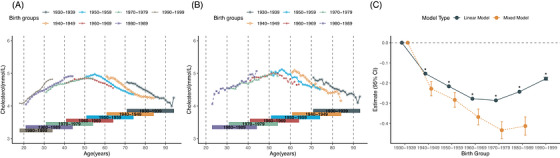
Analysis of total cholesterol levels with age and model analysis across different birth cohorts.

The unadjusted mean trend of TC with age showed that in the 1970s‒1990s birth cohorts, later birth cohorts had higher mean levels at the same age, while the opposite was true for the 1930s‒1960s birth cohorts: at the age of 31, the mean levels for the 1970s, 1980s and 1990s birth cohorts were 4.42 (.80), 4.52 (.87) and 4.74 (.93) mmol/L, respectively; while at the age of 71, the mean levels for the 1930s, 1940s and 1950s birth cohorts were 4.73 (1.05), 4.52 (1.11) and 4.42 (1.18) mmol/L, respectively (Figure 3A). After adjustment by linear regression model, TC levels in the overall population showed a gradual downward trend in the 1940s‒1960s birth cohorts, reaching the lowest point in the 1970s, and then the decline gradually rebounded (1940s: ‒.153 mmol/L; 1950s: ‒.216 mmol/L; 1960s: ‒.278 mmol/L; 1970s: ‒.286 mmol/L; 1980s: ‒.243 mmol/L; 1990s: ‒.178 mmol/L, all *p* <.001) (Figure 3C).

The trend in the fixed population was consistent with the overall population, with more noticeable fluctuations in the 1990s cohort due to the smaller sample size (Figure 3B). After adjustment by mixed‐effects model, the decline in TC was the greatest in the 1970s cohort, followed by a rebound (1940s: ‒.228 mmol/L; 1950s: ‒.284 mmol/L; 1960s: ‒.368 mmol/L; 1970s: ‒.433 mmol/L; 1980s: ‒.410 mmol/L, all *p* <.001) (Figure 3C).

### 3.4 Birth cohort effects on triglyceride levels

**FIGURE 4:**
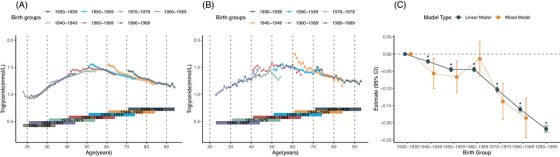
Analysis of triglyceride levels with age and model analysis across different birth cohorts.

In the overall population, individuals from later birth cohorts had lower TG levels than those from earlier birth cohorts. The unadjusted mean trend of TG with age showed that later birth cohorts had lower mean levels at the same age: at the age of 71 years, the geometric mean levels for the 1930s, 1940s and 1950s birth cohorts were 1.43, 1.35 and 1.32 mmol/L, respectively; while at the age of 31 years, the mean levels for the 1970s, 1980s and 1990s birth cohorts were 1.16, 1.15 and 1.13 mmol/L, respectively (Figure 4A). After adjustment by linear regression model (with the 1930s birth cohort as the reference group), TG levels in the overall population showed a gradual downward trend in the 1950s‒1960s birth cohorts, with a larger decline in the 1980s‒1990s birth cohorts (1940s: ‒.022 mmol/L; 1950s: ‒.045 mmol/L; 1960s: ‒.045 mmol/L; 1970s: ‒.103 mmol/L; 1980s: ‒.161 mmol/L; 1990s: ‒.218 mmol/L, all *p* <.001) (Figure 4C).

The trend in the fixed population was consistent with the overall population (Figure 4B). After adjustment by mixed‐effects model, the decline in TG was even greater in the 1990s birth cohort (1940s: ‒.057 mmol/L; 1950s: ‒.067 mmol/L; 1960s: ‒.014 mmol/L; 1970s: ‒.138 mmol/L; 1980s: ‒.185 mmol/L, all *p* <.001 except for the 1960s) (Figure 4C).

### 3.5 Birth cohort effects on low‐density lipoprotein cholesterol levels

**FIGURE 5:**
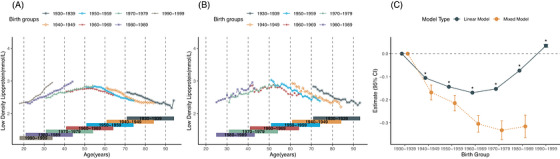
Analysis of low‐density lipoprotein cholesterol levels with age and model analysis across different birth cohorts.

LDL‐C levels showed a relatively consistent trend across birth cohorts. The unadjusted mean trend of LDL‐C with age showed that in the 1970s‒1990s birth cohorts, later birth cohorts had higher mean levels at the same age, while the opposite was true for the 1930s‒1960s birth cohorts: at the age of 31 years, the mean levels for the 1970s, 1980s and 1990s birth cohorts were 2.48 (.72), 2.54 (.77) and 2.81 (.81) mmol/L, respectively; while at the age of 71 years, the mean levels for the 1930s, 1940s and 1950s birth cohorts were 2.65 (.89), 2.50 (.92) and 2.45 (1.01) mmol/L, respectively (Figure 5A). Compared with TC, LDL‐C in the overall population also showed a downward trend followed by an upward trend in the 1980s‒1990s birth cohorts (1940s: ‒.105 mmol/L; 1950s: ‒.144 mmol/L; 1960s: ‒.169 mmol/L; 1970s: ‒.153 mmol/L; 1980s: ‒.074 mmol/L; 1990s: +.034 mmol/L, all *p* <.001) (Figure 5C).

The trend in the fixed population was consistent with the overall population, with more noticeable fluctuations in the 1990s cohort due to the smaller sample size (Figure 5B). After adjustment by mixed‐effects model, LDL‐C showed a continuous decline in the 1930s‒1970s cohorts, with a slight rebound in the 1980s cohort (1940s: ‒.168 mmol/L, *p* <.001; 1950s: ‒.215 mmol/L, *p* <.001; 1960s: ‒.304 mmol/L, *p* <.001; 1970s: ‒.332 mmol/L, *p* <.001; 1980s: ‒.311 mmol/L, *p* <.001) (Figure 5C).

### 3.6 Birth cohort effects on high‐density lipoprotein cholesterol levels

**FIGURE 6:**
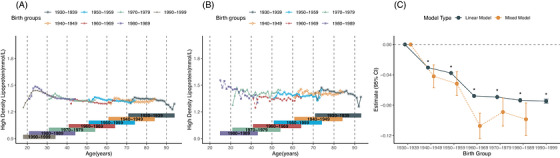
Analysis of high‐density lipoprotein cholesterol levels with age and model analysis across different birth cohorts.

In the overall population, individuals from later birth cohorts had lower HDL‐C levels than those from earlier birth cohorts. The unadjusted mean trend of HDL‐C with age showed that later birth cohorts had lower mean levels at the same age in most cases (Figure 6A). After adjustment by linear regression model (with the 1930s birth cohort as the reference group), HDL‐C levels in the overall population showed a continuous decline across birth cohorts, reaching the lowest point in the 1990s birth cohort (1940s: ‒.030 mmol/L; 1950s: ‒.038 mmol/L; 1960s: ‒.068 mmol/L; 1970s: ‒.069 mmol/L; 1980s: ‒.074 mmol/L; 1990s: ‒.075 mmol/L, all *p* <.001) (Figure 6C).

The trend in the fixed population was consistent with the overall population (Figure 6B). After adjustment by mixed‐effects model, HDL‐C levels showed a gradual decline in the 1930s‒1960s cohorts, with fluctuations but still lower values compared with the reference group (1940s: ‒.042 mmol/L; 1950s: ‒.052 mmol/L; 1960s: ‒.107 mmol/L; 1970s: ‒.089 mmol/L; 1980s: ‒.098 mmol/L) (Figure 6C).

### 3.7 Sensitivity analyses

To evaluate the robustness of our findings, we conducted sensitivity analyses by stratifying participants into health check‐up and patient subgroups. The results consistently demonstrated persistent birth cohort effects across all metabolic indicators (FG, TC, LDL‐C, HDL‐C and TG) in both subgroups (Figures S1‒S10). These effects remained statistically significant (*p* <  .05) in the majority of the overall population and fixed cohort models (Tables S2‒S11). Notably, while the direction of trends was similar between subgroups, the magnitude of changes varied. For instance, later‐born cohorts in health check‐ups showed larger increases in FG (e.g., +.46 mmol/L for 1990s vs. 1930s) compared to patients (+.39 mmol/L), whereas lipid changes (e.g., LDL‐C rebound in the 1980s‒1990s) were more pronounced in patients. These findings underscore the robustness of our primary conclusions and highlight population‐specific metabolic shifts, likely reflecting differences in lifestyle, healthcare access or underlying health status between subgroups. In addition, 5‐year intervals were explored to validate the robustness of the results, the results yielded consistent trends (Figures S11‒S15). The results of FG measuring methods change (before and after 2017) were also compared (Figures S16 and S17).

## 4 DISCUSSION

Against the backdrop of an increasing global burden of metabolic diseases and a significant trend of younger age, our study utilised real‐world clinical laboratory big data and conducted a large‐sample longitudinal analysis to depict for the first time the continuous change trajectories of metabolic indicators across Chinese birth cohorts, providing a basis for the early prevention and control of metabolic diseases.

Our study found significant birth cohort effects on metabolic indicators in the Chinese population. From the 1930s to the 1990s birth cohorts, individuals from later birth cohorts had higher FG levels than those from earlier birth cohorts, with the largest increase in the 1980s‒1990s cohorts. Compared with the 1930s birth cohort, the 1990s birth cohort had an increase of  .438 mmol/L in FG. Unlike the unidirectional increase in glucose, the lipid profile changes were more complex. TC and LDL‐C showed a non‐linear cohort effect, with a gradual increase in decline from the 1930s to the 1970s birth cohorts, followed by a rebound in the 1980s‒1990s cohorts. Compared with TC, the decline and rebound of LDL‐C were more pronounced; compared with the 1930s birth cohort, the 1990s birth cohort had an increase of  .034 mmol/L in LDL‐C. HDL‐C and TG showed a continuous decline, with decreases of  .075 and  .218 mmol/L, respectively, compared with the 1930s birth cohort. These metabolic shifts are clinically significant given their established roles in atherosclerosis and diabetes. Elevated LDL‐C drives atherosclerosis by infiltrating the arterial wall, becoming oxidised and triggering chronic inflammation. This leads to foam cell formation and, crucially, impaired clearance of these cells, which creates unstable plaques. Subsequent plaque rupture can cause thrombosis and acute events such as myocardial infarction.^11^ TG and their TG‐rich lipoprotein remnants promote arterial inflammation and cholesterol deposition.^12^ In contrast, HDL appears to exert beneficial effects on vascular calcification, although the regulatory mechanism of HDL for atherosclerosis is still not clear.^13^ Hyperglycaemia ultimately leads to diabetes through the dual mechanisms of aggravated insulin resistance and metabolic exhaustion of pancreatic β‐cells. Additionally, diabetes can accelerate the development process of atherosclerosis through turbulent blood flow, transformation of endothelial cells into mesenchymal cells, inflammation and oxidative stress. Moreover, hyperglycaemia inhibits NO production and stimulates the production of plasminogen activator inhibitor‐1.^14^, ^15^ Epidemiological evidence indicates that a 1 mmol/L reduction in LDL‐C is associated with a 21% lower risk of cardiovascular disease and a 12% reduction in all‐cause mortality.^16^ Conversely, each  .026 mmol/L increase in HDL‐C correlates with a 2%‒3% decrease in cardiovascular event risk.^17^ In addition, 1 mmol/L increase in FG above 5.3 mmol/L elevates cardiovascular and all‐cause mortality risk by 15%.^18^ Although the FBG levels of the 1980s‒1990s birth cohort are not yet critically high, their clear upward trajectory with age suggests that this generation may reach the risk‐significant threshold of 5.3 mmol/L at an earlier age in the future. Overall, individuals from later birth cohorts faced greater metabolic risks than those from earlier birth cohorts, especially those born in the 1980s‒1990s.

Compared with previous studies, our findings further corroborate the trend of earlier onset of abnormal glucose and lipid levels in the Chinese population. A study tracking the long‐term trends of glucose and lipid levels in the Chinese population from 1983 to 2011 showed that the standardised prevalence rates of high TG, low HDL‐C, dyslipidaemia, elevated FG and diabetes continued to rise.^19^ Another study found that compared with 2002, the 35 years and younger population had the largest increase in TC and TG, and the largest decrease in HDL‐C in 2010.^20^ Compared with other countries' data during the same period, the US population born from the 1920s to the 1990s had a continuous increase in FG levels (an increase of  .150 mmol/L every 10 years), while lipid levels improved (TC decreased by  .184 mmol/L every 10 years, and TG decreased by  .148 mmol/L every 10 years).^9^ This difference may be related to the significant difference in statin use rates between the two countries. In 2013, the US ACC/AHA guidelines expanded the scope of statin use, while the use rate of lipid‐lowering drugs in primary medical institutions in China was still low, although it had increased during the same period.^21^

In the earlier birth cohorts (1930s‒1970s), FG levels increased with birth cohorts, while TG, TC, LDL‐C and HDL‐C decreased. The inconsistent trends in glucose and lipids may be related to the dietary and lifestyle characteristics of that time. China was mainly in an agricultural economy stage, with a diet characterised by high carbohydrates and low fat (e.g., sweet potatoes and corn as staple foods). The increase in refined carbohydrate intake may have driven early insulin resistance.^22^ At the same time, the intake of animal fat and edible oil was extremely low, directly limiting the substrates for cholesterol and TG synthesis.^23^ On the other hand, early life factors, such as the material shortages during 1959‒1961, may have increased the risk of metabolic imbalance in later life for the 1930s‒1950s birth cohorts, but the early low‐fat diet still maintained low lipid levels.^24^ Additionally, the use of medications may have had an impact. As the main target of lipid‐lowering drugs, the significant decline in LDL‐C in the 1930s‒1970s birth cohorts may have been related to the increased use of statins,^25^ while HDL‐C, which has no effective drugs to increase, continued to decline generationally.

It is worth noting that in the later birth cohorts (1980s‒1990s), FG, TC and LDL‐C levels increased significantly, while HDL‐C levels decreased significantly. However, the observed decline in TG, despite rising glucose and LDL‐C, may be explained by several factors. First, TG levels are highly influenced by diet and physical activity and are readily metabolised, leading to significant fluctuations. In contrast, LDL‐C is regulated by LDL‐C receptors; once elevated, it is difficult to effectively clear without pharmacological intervention, which may explain their divergent trends. Furthermore, the evolution of modern dietary patterns towards higher protein and lower carbohydrate intake may also contribute by promoting TG reduction. This shift likely alters hepatic lipid metabolism and improves insulin sensitivity, thereby improving the overall metabolic profile.^26^ This unique metabolic shift in later cohorts may be the result of the combined effects of socioeconomic and behavioural factors. Over the past four decades, China's economic boom has been accompanied by a dramatic rise in urbanisation—from 17.9% to 66.2%. This shift brought westernised diets, sedentary behaviours and new daily stresses.^27^ Specifically, from 1982 to 2012, the modern high‐fat diet pattern gradually became popular in China, with meat consumption nearly tripling and edible oil intake nearly doubling.^23^, ^28^ This change in dietary structure is closely related to the increase in glucose and lipid levels.^29^, ^30^ Concurrently, from 2004 to 2018, the obesity rate increased rapidly from 3.1% to 8.1%.^31^ A study in China found that the most common complications among overweight/obese participants were fatty liver, prediabetes, dyslipidaemia and hypertension, and the number of complications increased with body mass index (BMI).^32^ In addition, with the process of urbanisation, the physical activity of the younger generation has decreased sharply, and a sedentary lifestyle increases the risk of cardiovascular diseases and diabetes.^33^ Moreover, smoking, drinking and staying up late are also important factors affecting metabolism.^34^ The later birth cohorts (1980s‒1990s) entered adulthood during a period of unprecedented economic growth and urbanisation, which shaped their dietary preferences, physical activity patterns and metabolic risks in ways that distinguish them from earlier generations.

In supplementary analyses, it is observed a steeper decline in older patient cohorts (1960s‒1970s) and a sharper rebound in younger check‐up cohorts (1980s‒1990s). This pattern suggests successful clinical intervention (e.g., statins) in managing established risks in older adults, which contrasts sharply with the unchecked detrimental impact of China's socioeconomic transition on the metabolic health of younger generations. This highlights a critical public health challenge: while healthcare effectively treats older patients, it fails to prevent risk accumulation in younger populations exposed to obesogenic environments.

Studies have shown that compared with the normal population, individuals who develop type 2 diabetes before the age of 40 years have a 3.72‐fold increased risk of death and higher risks of diabetes‐related complications, and poorer glucose control.^35^ A long‐term follow‐up of nearly 5000 young people showed that even if lipid levels were similar in middle age, those who had high lipids in their youth had a much higher risk of cardiovascular diseases such as myocardial infarction and stroke; and even after lipid control, there was still residual risk.^36^ However, recent studies have shown that abnormal glucose and lipid levels in young people are more reversible and can be effectively improved through weight loss, diet and medication.^37^, ^38^, ^39^, ^40^ These findings support the implementation of active interventions in young people to achieve the best metabolic outcomes. China's prevention and treatment guidelines have advanced the screening age for abnormal glucose in the general population to 35 years old, and for lipids to 40 years old. Based on the findings of our study, individuals from the later birth cohorts (1980s‒1990s) should receive earlier attention regarding their metabolic health, adopt changes in diet and lifestyle, and focus on preventing the onset of related diseases. Therefore, it is particularly important to conduct health education, early monitoring, and intervention for the younger generation to improve the prevention and control of metabolic abnormalities and enhance population health.

The strengths of this study include the following: first, we revealed for the first time the significant intergenerational differences in metabolic composite indicators in the Chinese population. Second, based on a large‐sample longitudinal cohort design, the long‐term continuous repeated measurement data of the fixed population effectively controlled for time variability and confounding factors, significantly enhancing the reliability of the conclusions, an advantage that cross‐sectional studies of the same kind lack. In addition, the study included multiple metabolic indicators such as FG, TC, LDL‐C, HDL‐C and TG, providing detailed data support for a comprehensive assessment of population metabolic characteristics.

The limitations of this study are as follows: first, the data of this study were derived from a large‐scale comprehensive hospital in Shanghai, which may limit generalisability to rural or other socioeconomic areas in China. Second, as a retrospective study based on the LIS database, the lack of individualised data (such as BMI, smoking, diet, physical activity, glycaemic history and medication records) limits the in‐depth analysis of factors influencing intergenerational differences. For instance, the observed secular trends in glucose and lipids could be partly attributed to generational differences in obesity prevalence, dietary patterns or the increasing use of medications in more recent birth cohorts. These factors may influence metabolic trends and should be addressed in future electronic health record analyses studies. Third, the 15‐year observation period did not fully cover the life cycle of each generation and the 1990s birth cohort within the fixed population was not analysed due to its small sample size, thus longer follow‐up is needed in the future. Finally, although four lipid tests were included, the complexity of lipid metabolism suggests that future studies should integrate other metabolic parameters and emerging biomarkers (such as sdLDL, Lp(a), etc.) for a comprehensive assessment.

For the first time, we revealed that there is a significant intergenerational effect on metabolic syndrome indicators in China. Overall, the younger generations, 1980s‒1990s cohorts, face more severe metabolic risks than those 1930s‒1970s cohorts. This trend may be related to the improvement of socioeconomic levels and changes in dietary structure. It is recommended to adopt multidimensional intervention strategies, including strengthening health policy supervision, improving preventive medication and promoting healthy diets and lifestyles, especially to improve the metabolic health of the younger generation.

## AUTHOR CONTRIBUTIONS

Ying Xiong, Xincen Duan and Jiayi Huang performed the statistical analyses and interpreted the data. Ying Xiong, Xincen Duan and Jing Zhu drafted the manuscript. Jing Zhu and Wenqi Shao contributed to the data acquisition and review for important intellectual content. Baishen Pan and Beili Wang revised the manuscript. Baishen Pan, Wei Guo and Beili Wang conceived and designed this study, and final version approval. All authors have read and approved the final manuscript. Beili Wang, Wei Guo and Wenqi Shao are the guarantors of this work and, as such, had full access to all the data in the study and take responsibility for the integrity of the data and the accuracy of the data analysis.

## CONFLICT OF INTEREST STATEMENT

The authors declare they have no conflicts of interest.

## ETHICS STATEMENT

The study was approved by the Ethics Committee of Fudan University Zhongshan Hospital (B2023‐363).

## Supporting information



Supporting information

## Data Availability

The data that support the findings of this study are available on request from the corresponding author. The data are not publicly available due to privacy or ethical restrictions.
