# Supplementary material for: Long‐term trends in metabolic profiles across Chinese birth cohorts: A real‐world big data analysis
Source: Clin Transl Med. 2026 Jun 17;16(6):e70660. doi: 10.1002/ctm2.70660 (PMC13276275; doi:10.1002/ctm2.70660)

**Supplementary materials**

**Part 1: Analysis of Participants Stratified into Health Check-up and Patient Subgroups** **(****S. Figure 1-10, S. Table 1-10)**

**Part 2: Analysis with a Smaller Interval of Five-year Intervals (S. Figure 11-15)**

**Part 3: Analysis of Fasting Glucose Measuring Methods Change (before and after 2017) (S. Figure 16-17)**

**Part 1: Analysis of Participants Stratified into Health Check-up and Patient Subgroups**

S. Figure 1. Analysis of Fasting Glucose Levels with Age and Model Analysis Across Different Birth Cohorts of health check-ups


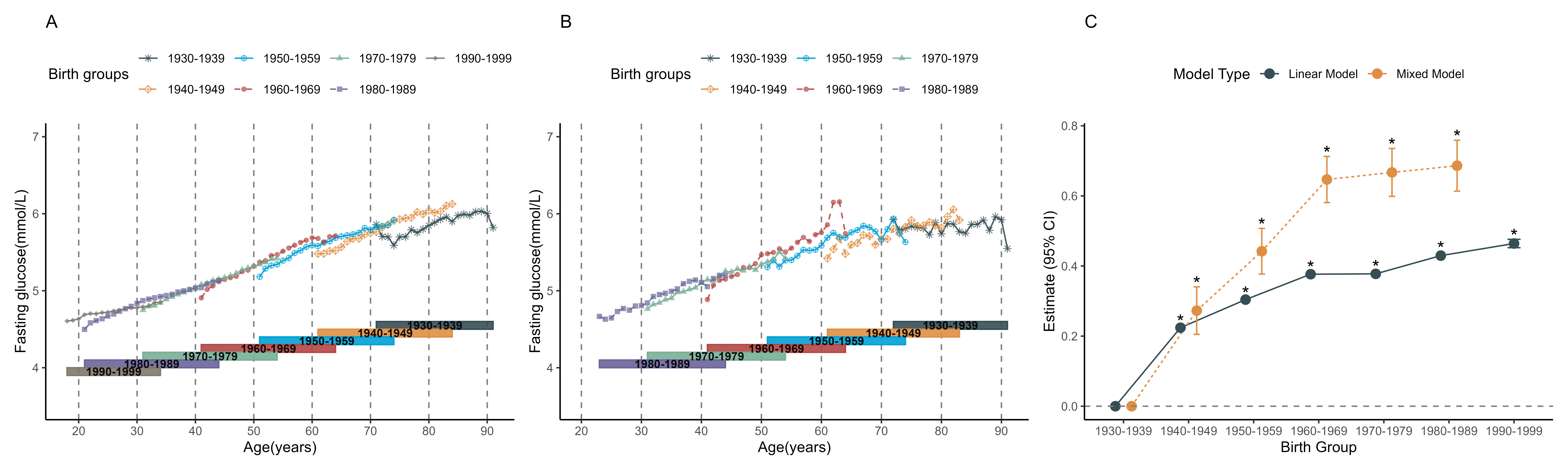


S. Figure 2. Analysis of Fasting Glucose Levels with Age and Model Analysis Across Different Birth Cohorts of patients


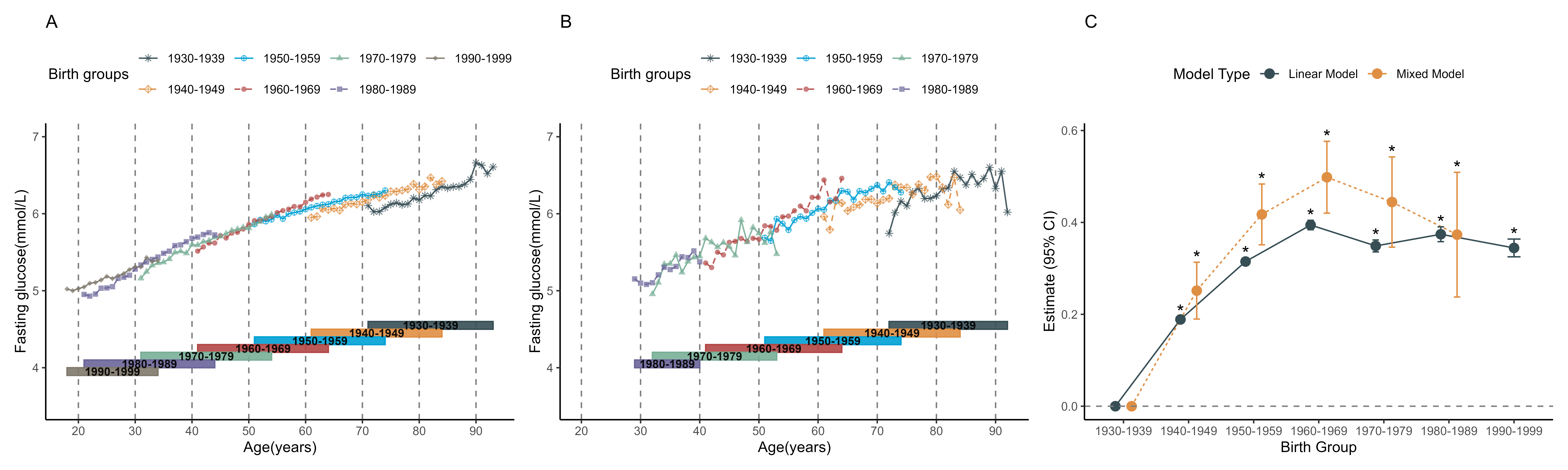


S. Figure 3. Analysis of Total Cholesterol Levels with Age and Model Analysis Across Different Birth Cohorts of health check-ups


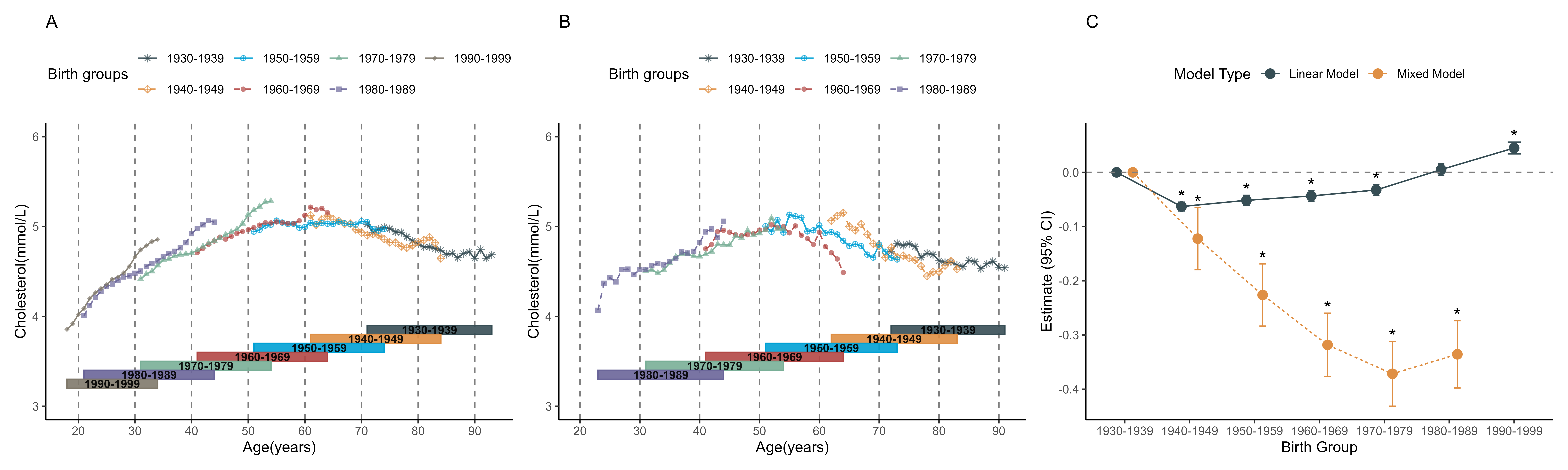


S. Figure 4. Analysis of Total Cholesterol Levels with Age and Model Analysis Across Different Birth Cohorts of patients


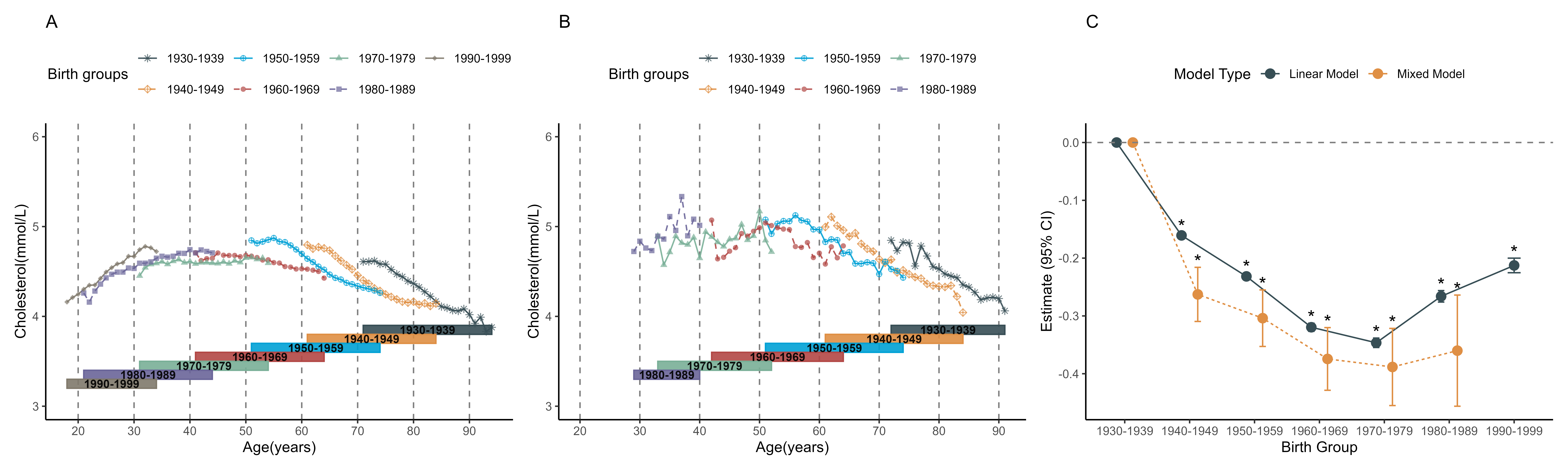


S. Figure 5. Analysis of Triglyceride Levels with Age and Model Analysis Across Different Birth Cohorts of health check-ups


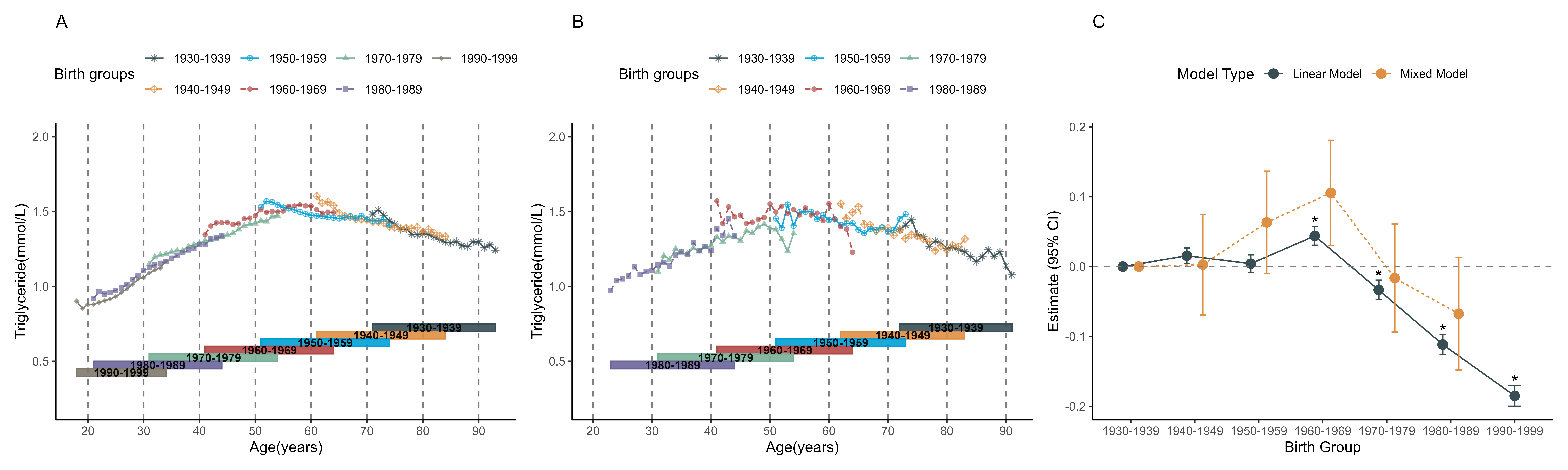


S. Figure 6. Analysis of Triglyceride Levels with Age and Model Analysis Across Different Birth Cohorts of patients


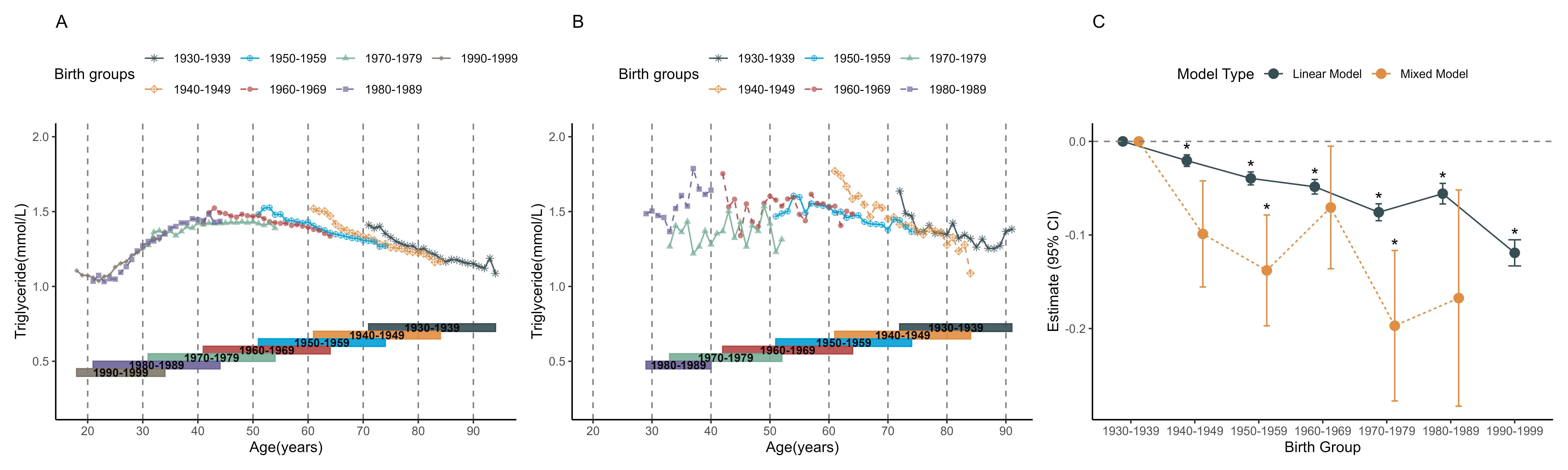


S. Figure 7. Analysis of Low-density Lipoprotein Cholesterol Levels with Age and Model Analysis Across Different Birth Cohorts of health check-ups


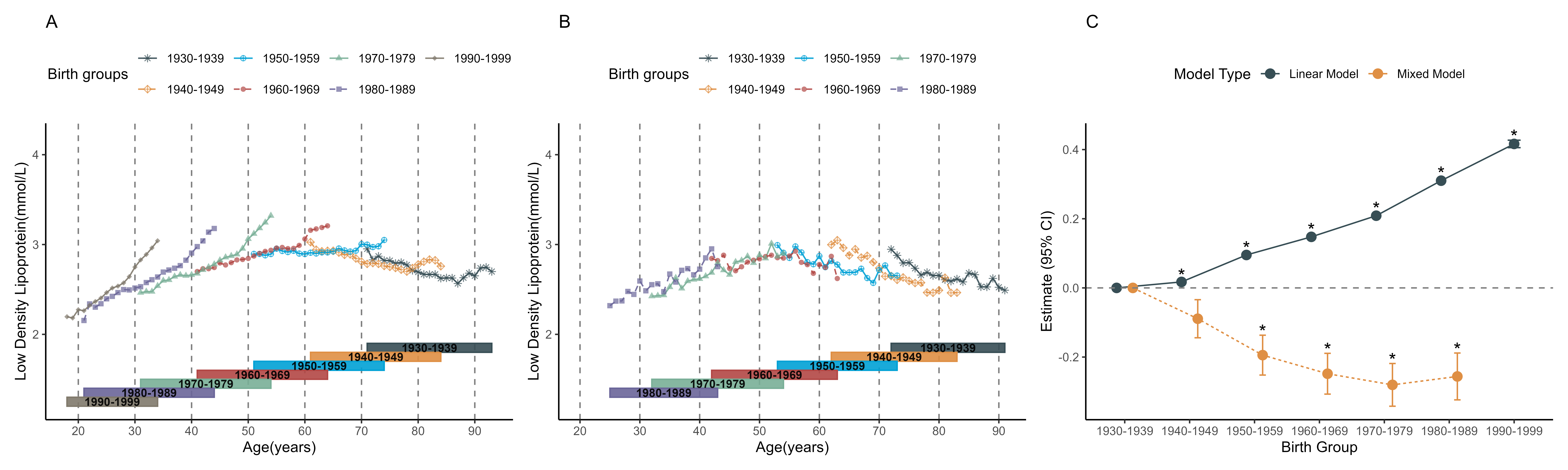


S. Figure 8. Analysis of Low-density Lipoprotein Cholesterol Levels with Age and Model Analysis Across Different Birth Cohorts of patients


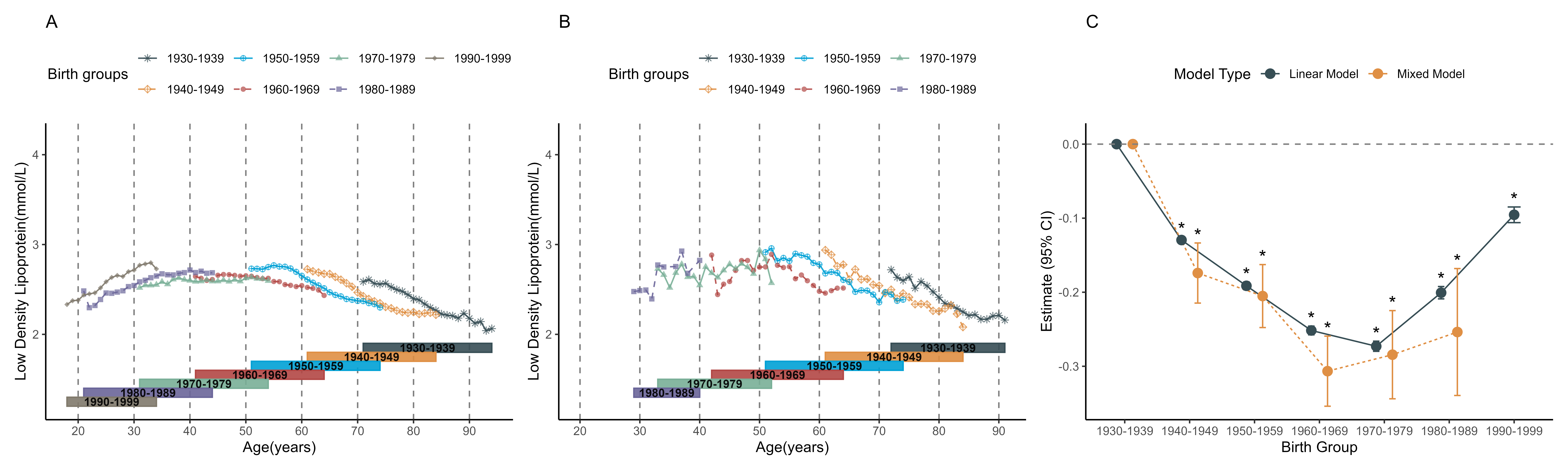


S. Figure 9. Analysis of High-density Lipoprotein Cholesterol Levels with Age and Model Analysis Across Different Birth Cohorts of health check-ups


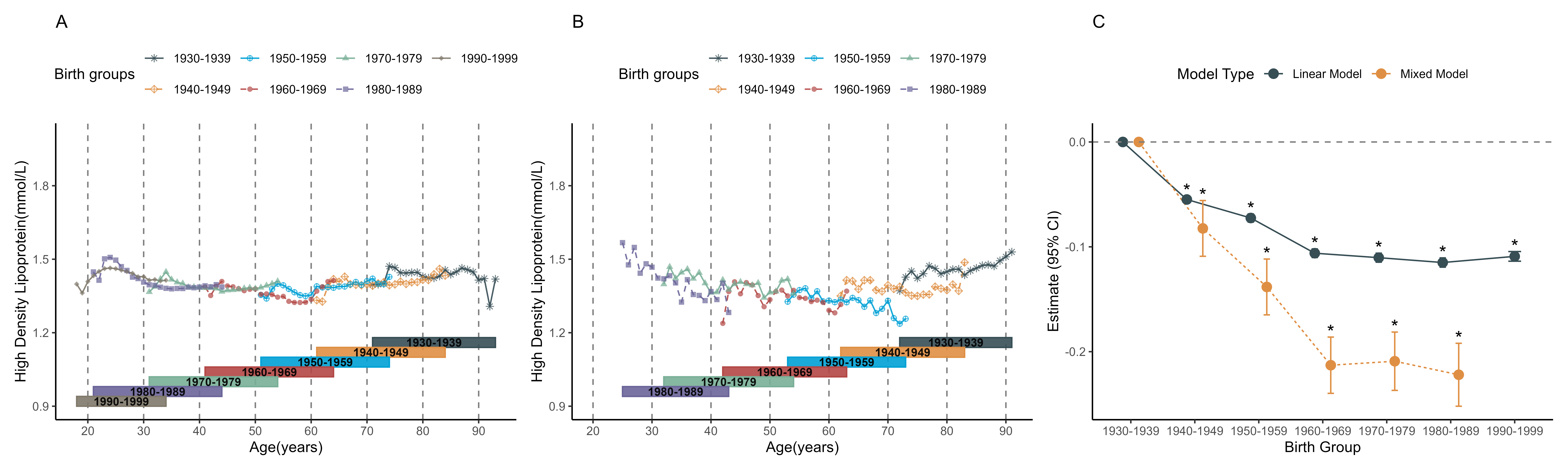


S. Figure 10. Analysis of High-density Lipoprotein Cholesterol Levels with Age and Model Analysis Across Different Birth Cohorts of patients


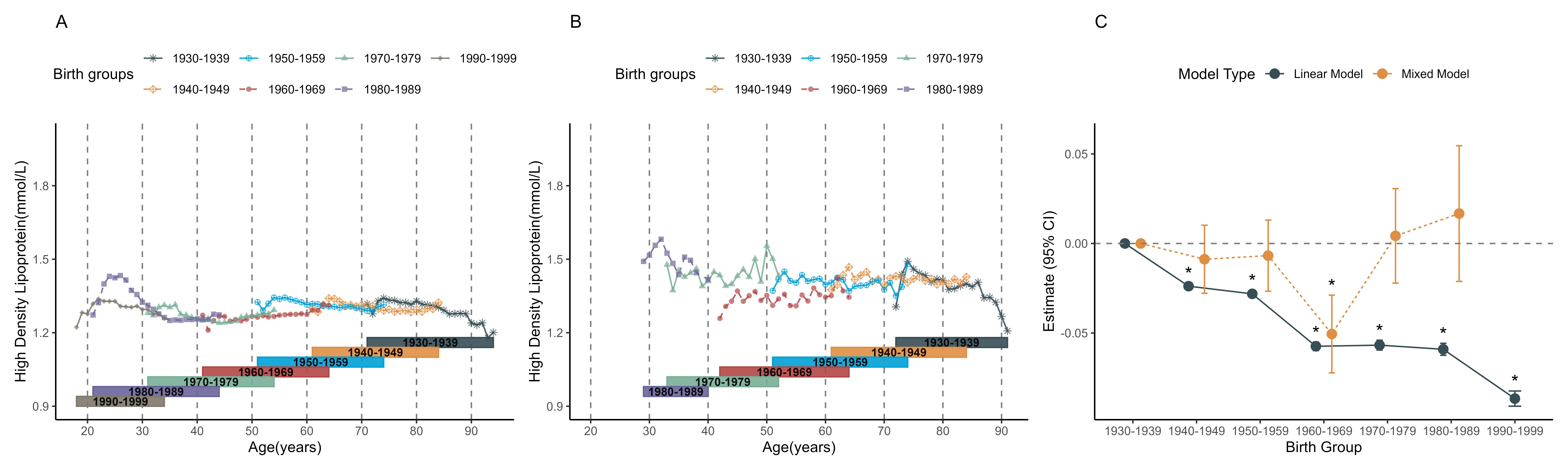


S. Table 1. Characteristics of the Study Population of Different Types of Participants

|  | Glucose | | Total cholesterol | | Low-density lipoprotein cholesterol | | High-density lipoprotein cholesterol | | Triglycerides | | ALL | |
| --- | --- | --- | --- | --- | --- | --- | --- | --- | --- | --- | --- | --- |
|  | Overall population | Fixed cohort | Overall population | Fixed cohort | Overall population | Fixed cohort | Overall population | Fixed cohort | Overall population | Fixed cohort | Overall population | Fixed cohort |
| Type |  |  |  |  |  |  |  |  |  |  |  |  |
| Patient | 1556862 (51.5) | 32685 (36.4) | 1284234 (47.6) | 26895 (42.3) | 1283608 (52.4) | 24930 (58.5) | 1281600 (52.4) | 24930 (58.7) | 1284733 (47.6) | 26715 (42.0) | 2221628 (57.1) | 65453 (46.3) |
| Health check-ups | 1468213 (48.5) | 57210 (63.6) | 1414413 (52.4) | 36690 (57.7) | 1164352 (47.6) | 17715 (41.5) | 1164464 (47.6) | 17505 (41.3) | 1414289 (52.4) | 36885 (58.0) | 1667233 (42.9) | 75872 (53.7) |

S. Table 2. The Results of the Fasting Glucose Birth Cohort Model for the health check-ups

|  | Model(Overall population) | | Model(Fixed cohort) | |
| --- | --- | --- | --- | --- |
| Birth groups | Estimate(95%CI) | P | Estimate(95%CI) | P |
| 1930-1939[Reference] | 0 |  | 0 |  |
| 1940-1949 | 0.22(0.216,0.232) | <0.001 | 0.27(0.205,0.34) | <0.001 |
| 1950-1959 | 0.3(0.297,0.312) | <0.001 | 0.44(0.377,0.507) | <0.001 |
| 1960-1969 | 0.38(0.368,0.385) | <0.001 | 0.65(0.581,0.713) | <0.001 |
| 1970-1979 | 0.38(0.368,0.387) | <0.001 | 0.67(0.599,0.735) | <0.001 |
| 1980-1989 | 0.43(0.419,0.441) | <0.001 | 0.69(0.614,0.759) | <0.001 |
| 1990-1999 | 0.46(0.452,0.476) | <0.001 |  |  |

S. Table 3. The Results of the Fasting Glucose Birth Cohort Model for the patients

|  | **Model(Overall population)** | | **Model(Fixed cohort)** | |
| --- | --- | --- | --- | --- |
| Birth groups | Estimate(95%CI) | P | Estimate(95%CI) | P |
| 1930-1939[Reference] | 0 |  | 0 |  |
| 1940-1949 | 0.19(0.182,0.196) | <0.001 | 0.25(0.19,0.313) | <0.001 |
| 1950-1959 | 0.31(0.307,0.323) | <0.001 | 0.42(0.351,0.484) | <0.001 |
| 1960-1969 | 0.39(0.384,0.405) | <0.001 | 0.5(0.42,0.577) | <0.001 |
| 1970-1979 | 0.35(0.336,0.362) | <0.001 | 0.44(0.346,0.543) | <0.001 |
| 1980-1989 | 0.37(0.358,0.39) | <0.001 | 0.37(0.238,0.509) | 0.006 |
| 1990-1999 | 0.34(0.325,0.364) | <0.001 |  |  |

S. Table 4. The Results of the Total Cholesterol Birth Cohort Model for the health check-ups

|  | **Model(Overall population)** | | **Model(Fixed cohort)** | |
| --- | --- | --- | --- | --- |
| Birth groups | Estimate(95%CI) | P | Estimate(95%CI) | P |
| 1930-1939[Reference] | 0 |  | 0 |  |
| 1940-1949 | -0.06(-0.071,-0.055) | <0.001 | -0.12(-0.18,-0.065) | 0.033 |
| 1950-1959 | -0.05(-0.061,-0.042) | <0.001 | -0.23(-0.284,-0.169) | <0.001 |
| 1960-1969 | -0.04(-0.053,-0.034) | <0.001 | -0.32(-0.377,-0.26) | <0.001 |
| 1970-1979 | -0.03(-0.043,-0.022) | 0.001 | -0.37(-0.431,-0.312) | <0.001 |
| 1980-1989 | 0.01(-0.005,0.015) | 0.623 | -0.34(-0.398,-0.273) | <0.001 |
| 1990-1999 | 0.04(0.034,0.056) | <0.001 |  |  |

S. Table 5. The Results of the Total Cholesterol Birth Cohort Model for the patients

|  | **Model(Overall population)** | | **Model(Fixed cohort)** | |
| --- | --- | --- | --- | --- |
| Birth groups | Estimate(95%CI) | P | Estimate(95%CI) | P |
| 1930-1939[Reference] | 0 |  | 0 |  |
| 1940-1949 | -0.16(-0.166,-0.155) | <0.001 | -0.26(-0.31,-0.216) | <0.001 |
| 1950-1959 | -0.23(-0.238,-0.225) | <0.001 | -0.3(-0.353,-0.255) | <0.001 |
| 1960-1969 | -0.32(-0.327,-0.313) | <0.001 | -0.37(-0.429,-0.32) | <0.001 |
| 1970-1979 | -0.35(-0.355,-0.338) | <0.001 | -0.39(-0.455,-0.322) | <0.001 |
| 1980-1989 | -0.27(-0.276,-0.256) | <0.001 | -0.36(-0.456,-0.264) | <0.001 |
| 1990-1999 | -0.21(-0.225,-0.2) | <0.001 |  |  |

S. Table 6. The Results of the Triglyceride Birth Cohort Model for the health check-ups

|  | Model(Overall population) | | Model(Fixed cohort) | |
| --- | --- | --- | --- | --- |
| Birth groups | Estimate(95%CI) | P | Estimate(95%CI) | P |
| 1930-1939[Reference] | 0 |  | 0 |  |
| 1940-1949 | 0.02(0.005,0.027) | 0.160 | 0(-0.069,0.075) | 0.969 |
| 1950-1959 | 0(-0.009,0.017) | 0.740 | 0.06(-0.01,0.137) | 0.390 |
| 1960-1969 | 0.04(0.031,0.057) | 0.001 | 0.11(0.03,0.181) | 0.160 |
| 1970-1979 | -0.03(-0.047,-0.019) | 0.017 | -0.02(-0.094,0.061) | 0.831 |
| 1980-1989 | -0.11(-0.126,-0.097) | <0.001 | -0.07(-0.148,0.013) | 0.403 |
| 1990-1999 | -0.18(-0.2,-0.17) | <0.001 |  |  |

S. Table 7. The Results of the Triglyceride Birth Cohort Model for the patients

|  | Model(Overall population) | | Model(Fixed cohort) | |
| --- | --- | --- | --- | --- |
| Birth groups | Estimate(95%CI) | P | Estimate(95%CI) | P |
| 1930-1939[Reference] | 0 |  | 0 |  |
| 1940-1949 | -0.02(-0.027,-0.014) | 0.001 | -0.1(-0.156,-0.042) | 0.081 |
| 1950-1959 | -0.04(-0.046,-0.033) | <0.001 | -0.14(-0.197,-0.079) | 0.020 |
| 1960-1969 | -0.05(-0.056,-0.041) | <0.001 | -0.07(-0.136,-0.005) | 0.281 |
| 1970-1979 | -0.08(-0.085,-0.067) | <0.001 | -0.2(-0.277,-0.117) | 0.014 |
| 1980-1989 | -0.06(-0.067,-0.045) | <0.001 | -0.17(-0.283,-0.052) | 0.147 |
| 1990-1999 | -0.12(-0.133,-0.105) | <0.001 |  |  |

S. Table 8. The Results of the Low-density Lipoprotein Cholesterol Birth Cohort Model for the health check-ups

|  | Model(Overall population) | | Model(Fixed cohort) | |
| --- | --- | --- | --- | --- |
| Birth groups | Estimate(95%CI) | P | Estimate(95%CI) | P |
| 1930-1939[Reference] | 0 |  | 0 |  |
| 1940-1949 | 0.02(0.009,0.025) | 0.030 | -0.09(-0.144,-0.034) | 0.106 |
| 1950-1959 | 0.1(0.086,0.104) | <0.001 | -0.19(-0.252,-0.137) | 0.001 |
| 1960-1969 | 0.15(0.138,0.157) | <0.001 | -0.25(-0.307,-0.189) | <0.001 |
| 1970-1979 | 0.21(0.199,0.219) | <0.001 | -0.28(-0.342,-0.219) | <0.001 |
| 1980-1989 | 0.31(0.3,0.32) | <0.001 | -0.26(-0.324,-0.188) | <0.001 |
| 1990-1999 | 0.42(0.406,0.427) | <0.001 |  |  |

S. Table 9. The Results of the Low-density Lipoprotein Cholesterol Birth Cohort Model for the patients

|  | Model(Overall population) | | Model(Fixed cohort) | |
| --- | --- | --- | --- | --- |
| Birth groups | Estimate(95%CI) | P | Estimate(95%CI) | P |
| 1930-1939[Reference] | 0 |  | 0 |  |
| 1940-1949 | -0.13(-0.134,-0.125) | <0.001 | -0.17(-0.215,-0.133) | <0.001 |
| 1950-1959 | -0.19(-0.197,-0.186) | <0.001 | -0.21(-0.248,-0.163) | <0.001 |
| 1960-1969 | -0.25(-0.258,-0.246) | <0.001 | -0.31(-0.354,-0.259) | <0.001 |
| 1970-1979 | -0.27(-0.28,-0.266) | <0.001 | -0.28(-0.344,-0.225) | <0.001 |
| 1980-1989 | -0.2(-0.209,-0.192) | <0.001 | -0.25(-0.339,-0.168) | 0.003 |
| 1990-1999 | -0.1(-0.106,-0.085) | <0.001 |  |  |

S. Table 10. The Results of the High-density Lipoprotein Cholesterol Birth Cohort Model for the health check-ups

|  | Model(Overall population) | | Model(Fixed cohort) | |
| --- | --- | --- | --- | --- |
| Birth groups | Estimate(95%CI) | P | Estimate(95%CI) | P |
| 1930-1939[Reference] | 0 |  | 0 |  |
| 1940-1949 | -0.05(-0.058,-0.051) | <0.001 | -0.08(-0.109,-0.056) | 0.002 |
| 1950-1959 | -0.07(-0.076,-0.069) | <0.001 | -0.14(-0.165,-0.112) | <0.001 |
| 1960-1969 | -0.11(-0.11,-0.102) | <0.001 | -0.21(-0.24,-0.186) | <0.001 |
| 1970-1979 | -0.11(-0.115,-0.106) | <0.001 | -0.21(-0.237,-0.181) | <0.001 |
| 1980-1989 | -0.11(-0.119,-0.111) | <0.001 | -0.22(-0.252,-0.192) | <0.001 |
| 1990-1999 | -0.11(-0.114,-0.104) | <0.001 |  |  |

S. Table 11. The Results of the High-density Lipoprotein Cholesterol Birth Cohort Model for the patients

|  | Model(Overall population) | | Model(Fixed cohort) | |
| --- | --- | --- | --- | --- |
| Birth groups | Estimate(95%CI) | P | Estimate(95%CI) | P |
| 1930-1939[Reference] | 0 |  | 0 |  |
| 1940-1949 | -0.02(-0.026,-0.022) | <0.001 | -0.01(-0.028,0.01) | 0.643 |
| 1950-1959 | -0.03(-0.03,-0.026) | <0.001 | -0.01(-0.027,0.013) | 0.733 |
| 1960-1969 | -0.06(-0.06,-0.055) | <0.001 | -0.05(-0.072,-0.029) | 0.020 |
| 1970-1979 | -0.06(-0.06,-0.054) | <0.001 | 0(-0.022,0.031) | 0.872 |
| 1980-1989 | -0.06(-0.062,-0.056) | <0.001 | 0.02(-0.021,0.055) | 0.659 |
| 1990-1999 | -0.09(-0.091,-0.082) | <0.001 |  |  |

**Part 2: Analysis with a Smaller Interval of Five-year Intervals**

S. Figure 11. Analysis of Fasting Glucose Levels with Age and Model Analysis of Five-year Intervals


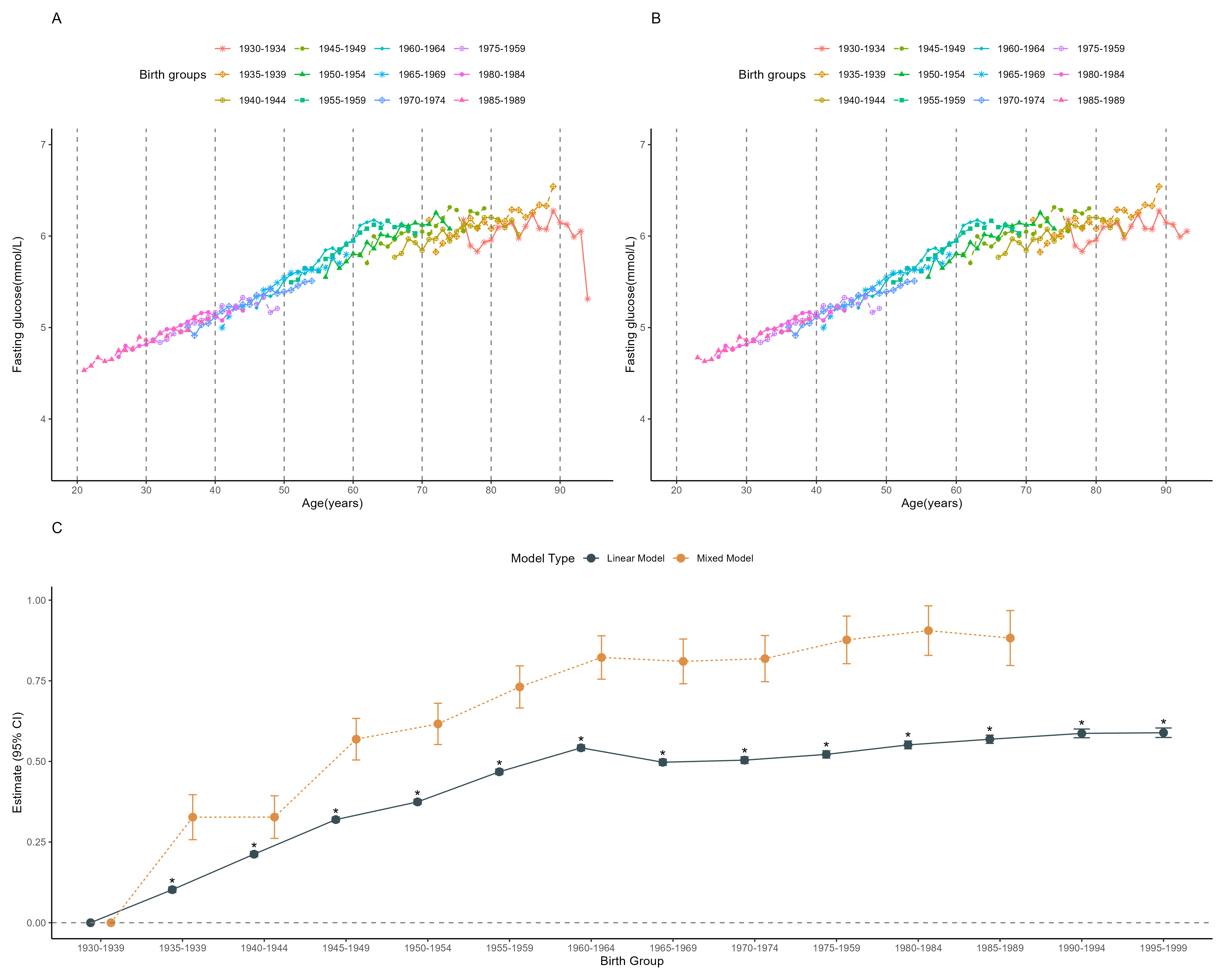


S. Figure 12. Analysis of Total Cholesterol Levels with Age and Model Analysis of Five-year Intervals


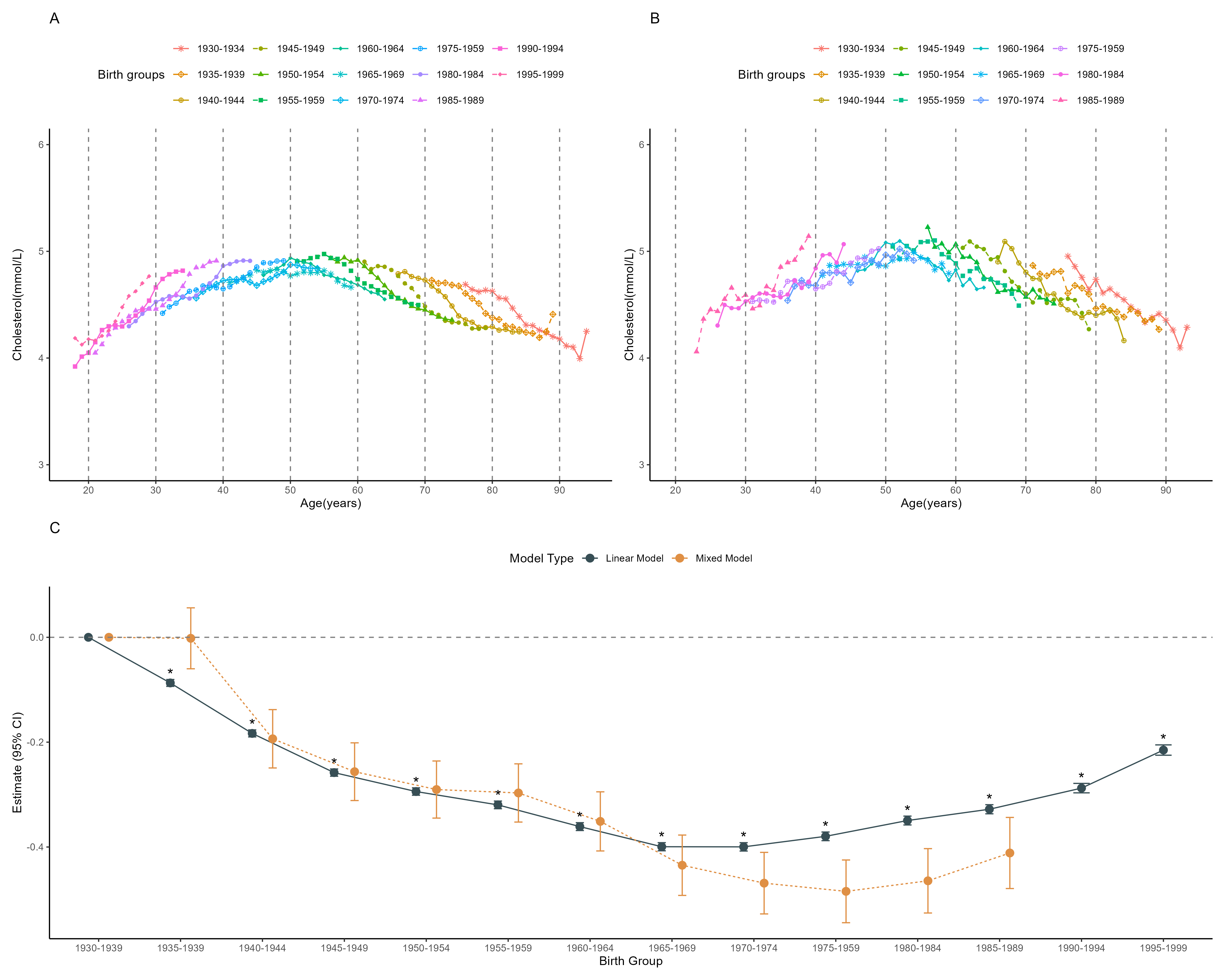


S. Figure 13. Analysis of Triglyceride Levels with Age and Model Analysis of Five-year Intervals


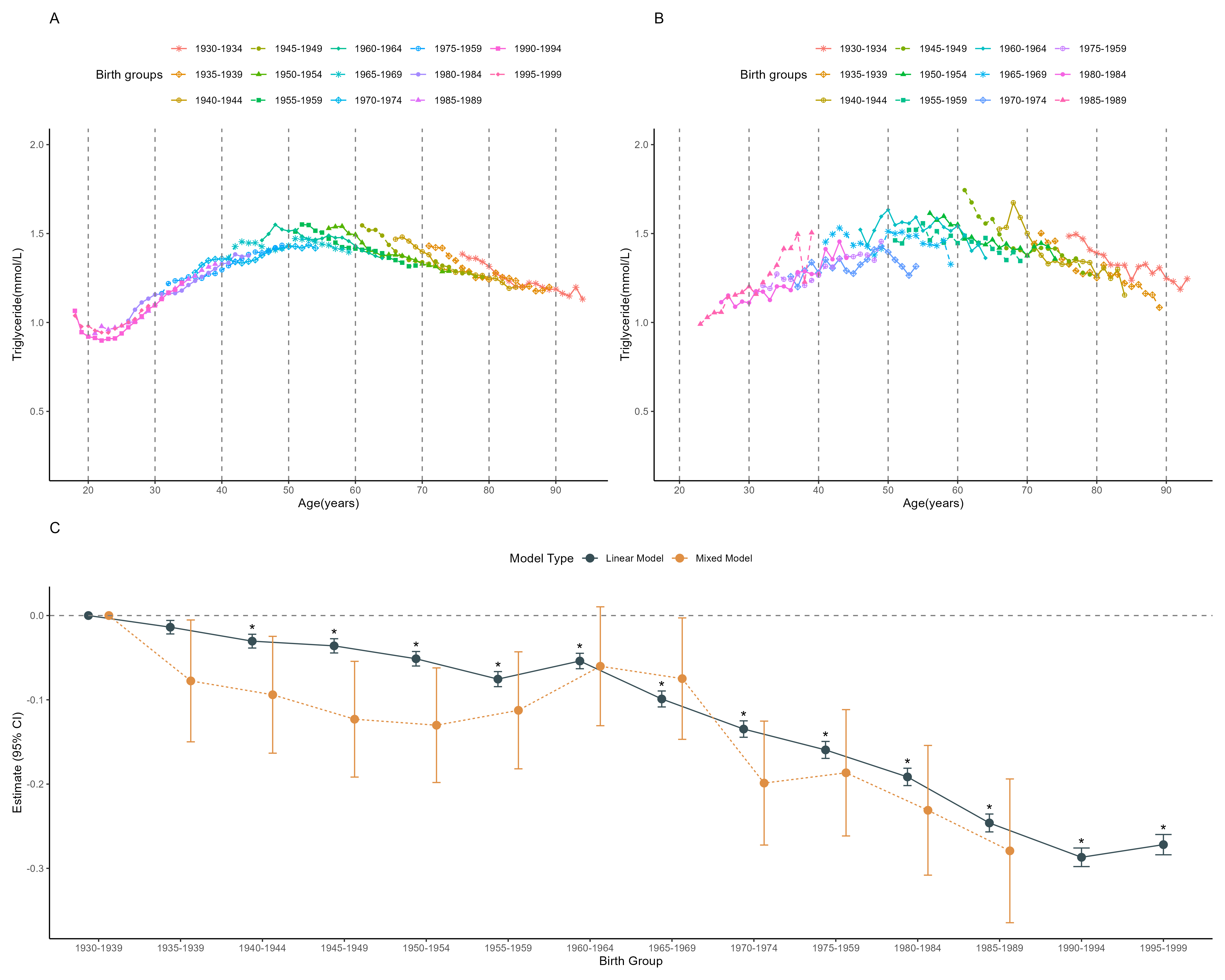


S. Figure 14. Analysis of Low-density Lipoprotein Cholesterol Levels with Age and Model Analysis of Five-year Intervals


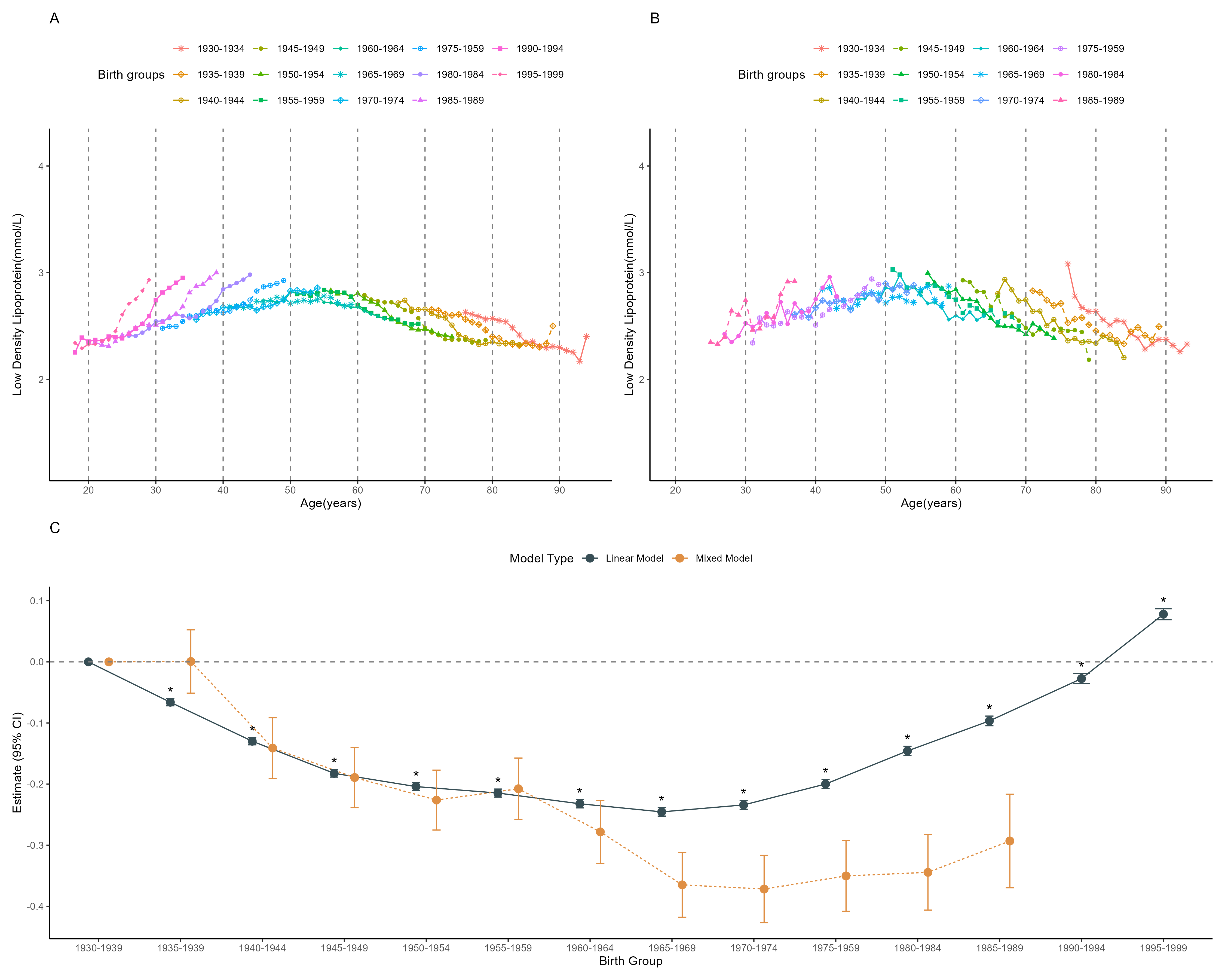


S. Figure 15. Analysis of High-density Lipoprotein Cholesterol Levels with Age and Model Analysis of Five-year Intervals


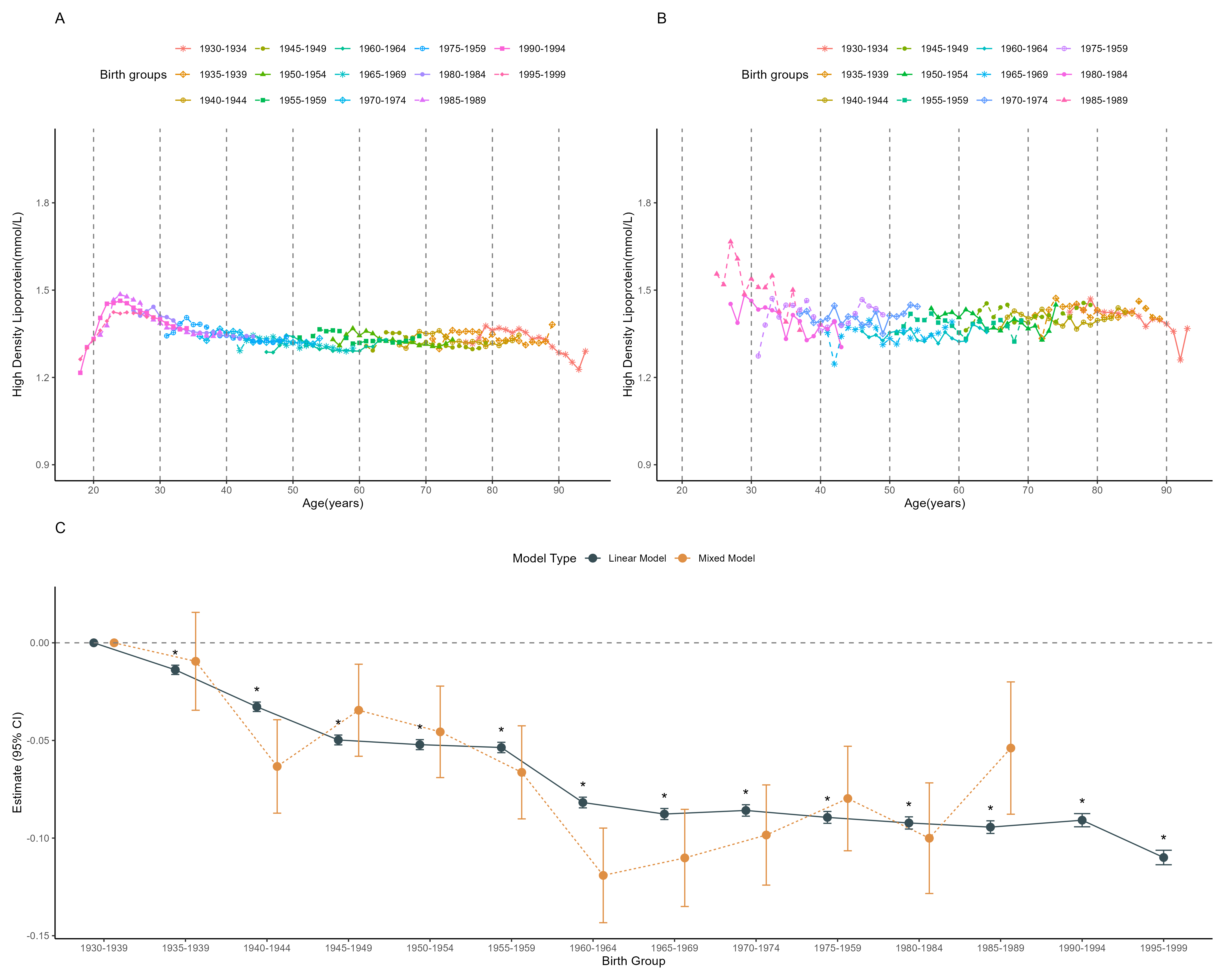


**Part 3: Analysis of Fasting Glucose Measuring Methods Change (before and after 2017)**

S. Figure 16. Results of Glucose Oxidase Method (before 2017)


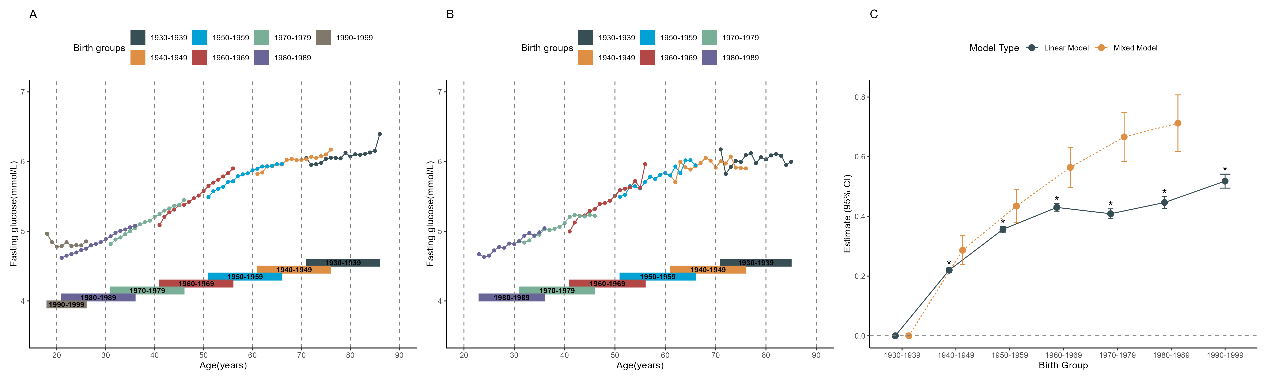


S. Figure 17. Results of Hexokinase Method (after 2017)


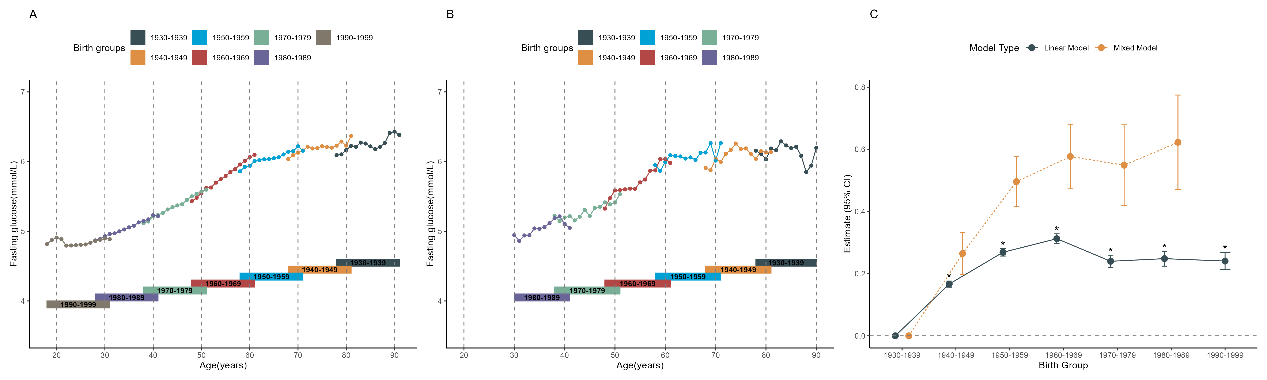

Supplement: Supplementary file 1 — Supporting information [file CTM2-16-e70660-s001.docx]
